# Biallelic variants in *YRDC* cause a developmental disorder with progeroid features

**DOI:** 10.1007/s00439-021-02347-3

**Published:** 2021-09-20

**Authors:** Julia Schmidt, Jonas Goergens, Tatiana Pochechueva, Annika Kotter, Niko Schwenzer, Maren Sitte, Gesa Werner, Janine Altmüller, Holger Thiele, Peter Nürnberg, Jörg Isensee, Yun Li, Christian Müller, Barbara Leube, H. Christian Reinhardt, Tim Hucho, Gabriela Salinas, Mark Helm, Ron D. Jachimowicz, Dagmar Wieczorek, Tobias Kohl, Stephan E. Lehnart, Gökhan Yigit, Bernd Wollnik

**Affiliations:** 1 Institute of Human Genetics, University Medical Center Göttingen, Heinrich-Düker-Weg 12, 37073 Göttingen, Germany; 2 Max-Planck-Institute for Biology of Ageing, Cologne, Germany; 3 Heart Research Center Göttingen, Department of Cardiology and Pneumology, University Medical Center Göttingen, Göttingen, Germany; 4 Institute of Pharmaceutical and Biomedical Sciences, Johannes Gutenberg-University Mainz, Mainz, Germany; 5 NGS-Integrative Genomics Core Unit (NIG), Institute of Human Genetics, University Medical Center Göttingen, Göttingen, Germany; 6 Cologne Center for Genomics (CCG), University of Cologne, Faculty of Medicine and University Hospital Cologne, Cologne, Germany; 7 Institute of Human Genetics, Medical Faculty, Heinrich-Heine-University, Düsseldorf, Germany; 8 Department of Hematology and Stem Cell Transplantation, University Hospital Essen, University Duisburg-Essen, German Cancer Consortium (DKTK Partner Site Essen), Essen, Germany; 9 Department of Anesthesiology and Intensive Care Medicine, Translational Pain Research, University Hospital of Cologne, Cologne, Germany; 10 Department I of Internal Medicine, Faculty of Medicine and University Hospital Cologne, Cologne, Germany; 11 Cologne Excellence Cluster on Cellular Stress Response in Aging-Associated Diseases, University of Cologne, Cologne, Germany; 12 Center for Molecular Medicine Cologne, University of Cologne, Cologne, Germany; 13 DZHK (German Centre for Cardiovascular Research), partner site, Göttingen, Germany; 14 Collaborative Research Unit SFB 1002, University of Göttingen, Göttingen, Germany; 15 Cluster of Excellence “Multiscale Bioimaging: From Molecular Machines to Networks of Excitable Cells” (MBExC), University of Göttingen, Göttingen, Germany; 16 Berlin Institute of Health at Charité – Universitätsmedizin Berlin, Core Facility Genomics, Charitéplatz 1, 10117 Berlin, Germany; 17 Max Delbrück Center for Molecular Medicine in the Helmholtz Association (MDC), Berlin, Germany; 18 Collaborative Research Unit SFB 1190, University of Göttingen, Göttingen, Germany; 19 Transatlantic Network of Excellence CURE-PLaN, Fondation Leducq, Paris, France

## Abstract

The highly conserved YrdC domain-containing protein (YRDC) interacts with the well-described KEOPS complex, regulating specific tRNA modifications to ensure accurate protein synthesis. Previous studies have linked the KEOPS complex to a role in promoting telomere maintenance and controlling genome integrity. Here, we report on a newborn with a severe neonatal progeroid phenotype including generalized loss of subcutaneous fat, microcephaly, growth retardation, wrinkled skin, renal failure, and premature death at the age of 12 days. By trio whole-exome sequencing, we identified a novel homozygous missense mutation, c.662T > C, in *YRDC* affecting an evolutionary highly conserved amino acid (p.Ile221Thr). Functional characterization of patient-derived dermal fibroblasts revealed that this mutation impairs YRDC function and consequently results in reduced t^6^A modifications of tRNAs. Furthermore, we established and performed a novel and highly sensitive 3-D Q-FISH analysis based on single-telomere detection to investigate the impact of YRDC on telomere maintenance. This analysis revealed significant telomere shortening in YRDC-mutant cells. Moreover, single-cell RNA sequencing analysis of YRDC-mutant fibroblasts revealed significant transcriptome-wide changes in gene expression, specifically enriched for genes associated with processes involved in DNA repair. We next examined the DNA damage response of patient’s dermal fibroblasts and detected an increased susceptibility to genotoxic agents and a global DNA double-strand break repair defect. Thus, our data suggest that YRDC may affect the maintenance of genomic stability. Together, our findings indicate that biallelic variants in *YRDC* result in a developmental disorder with progeroid features and might be linked to increased genomic instability and telomere shortening.

**Supplementary Information:**

The online version contains supplementary material available at 10.1007/s00439-021-02347-3.

## Introduction

Segmental progeroid syndromes are rare congenital disorders characterized by signs and symptoms of premature or accelerated aging. Typical features of progeroid syndromes include, e.g., lipodystrophy, growth retardation, hair loss, brittle bones, atherosclerosis, and hearing loss. Several of these progeroid disorders show overlapping phenotypes, which makes specific clinical diagnosis often challenging. Most progeroid syndromes known so far belong to a very heterogeneous group of disorders caused by autosomal dominantly or recessively inherited mutations in a single gene. Yet identified cellular pathways and molecular pathomechanisms underlying premature aging mainly affect DNA damage repair processes, nuclear membrane dynamics, chromatin structure or transcription and, thereby, have an impact on various aspects of cell viability (Gordon et al. 2014; Carrero et al. 2016). Genomic instability as well as telomere shortening have been identified as particularly relevant for aging-associated processes (Harley et al. 1990; Blackburn 1991; Lange 2005; Martínez and Blasco 2011). In many aspects, the molecular characteristics of progeroid syndromes seem to be similar to those of “physiological” aging. Therefore, studying conditions of premature aging will help to reveal unknown underlying causal mechanisms and to develop potentially new treatments for more frequent age-associated diseases (Lessel and Kubisch 2019).

Previous studies suggest that YRDC (YrdC domain-containing protein; OMIM#612276), the human homolog of yeast Sua5, contributes to various central cellular functions. First, YRDC has been described to be necessary for adding specific tRNA modifications that are essential to ensure the accuracy of protein synthesis. YRDC in combination with the Kinase, Endopeptidase and Other Proteins of small Size (KEOPS) protein complex synthesizes the universally conserved threonylcarbamoylation of the N6 nitrogen of the adenosine at the tRNA position 37 (t^6^A) (Lin et al. 2018). Mutations in genes encoding subunits of the KEOPS complex lead to decreased t^6^A levels and cause Galloway–Mowat syndrome (GAMOS, OMIM#251300), a rare autosomal recessive condition characterized by the association of early onset nephrotic syndrome and microcephaly with central nervous system anomalies (Braun et al. 2017). Second, the KEOPS complex and Sua5 have been identified to promote telomere maintenance, an essential process for safeguarding genomic stability (Downey et al. 2006; Meng et al. 2010). Third, He et al*.* (2019) identified that the KEOPS complex affects both DNA damage response (DDR) and repair, especially homologous recombination-mediated DNA repair, independently of its t^6^A synthesis function. Very recently, two missense mutations in *YRDC* were associated with GAMOS phenotypes, suggesting overlapping functional roles of YRDC and the KEOPS complex in cellular processes (Arrondel et al. 2019).

Here, we report on a patient with a severe developmental disorder with progeroid features in whom we identified a novel causative, homozygous *YRDC* mutation. Analysis of fibroblasts revealed reduced levels of tRNA modification, telomere shortening, and impaired DDR.

## Results

### Clinical report

**Fig. 1:**
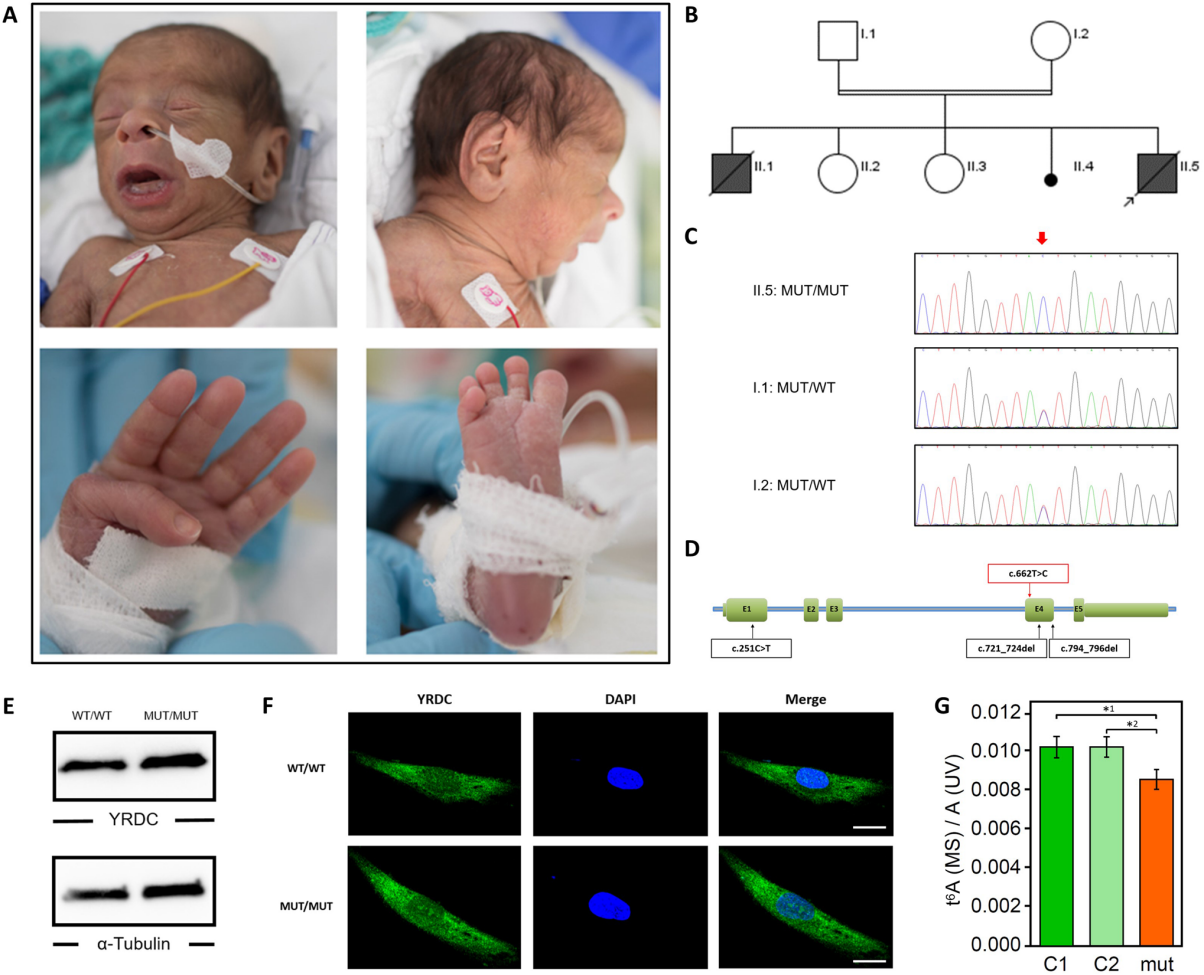
Identification of a homozygous mutation in *YRDC* in a newborn with a progeroid phenotype. **A** Clinical characteristics of the index patient II.5 at the age of 5 days. Physical features included generalized loss of subcutaneous fat with a progeroid appearance, hypertrichosis, low-set and large appearing ears, micro- and retrognathia, long, smooth philtrum, wrinkled skin, arachnodactyly and adducted thumbs. **B** Family pedigree of the consanguineous family from northern Iraq. Index patient is indicated by arrow. **C** Electropherograms of the identified *YRDC* mutation (red arrow) confirming homozygous state in the affected sibling II.5 and heterozygous carrier status of both parents, I.1 and I.2 (*MUT* mutation, *WT* wild type). **D** Schematic view of *YRDC* and localization of the identified mutations. Red arrow indicates the *YRDC* mutation identified within this study, previously identified mutations are indicated by black arrows. **E** Western blot analysis was performed on whole-cell lysates from healthy control fibroblasts (WT/WT) and patient (II.5)-derived fibroblasts (MUT/MUT) using anti-YRDC antibody (upper panel). Anti-α-Tubulin antibody was used as a loading control (lower panel). **F** Analysis of subcellular localization of WT (upper panel) and mutant (lower panel) YRDC by immunofluorescence staining in patient-derived (MUT) and control (WT) fibroblasts using anti-YRDC-antibodies (green). Nuclei were counterstained with DAPI (blue). Scale bar, 20 µm. **G** LC*–*MS/MS-based quantification of t^6^A levels in patient-derived fibroblasts (MUT) and two different controls (C1 and C2). Technical triplicates have been performed on one biological sample each. Levels are shown as MS signal of t^6^A divided by UV signal of adenosine. *p* values were calculated using two-sided *t* test: *^1^*p* = 0.0285, *^2^*p* = 0.0259

The index patient II.5 was born after 37 + 0 weeks of gestation to healthy, distantly consanguineous parents from northern Iraq origin (Fig. 1). His birth weight was 1740 g (− 3.1 SD), his birth length 46 cm (− 1.8 SD), and his head circumference (OFC) at birth 29 cm (− 3.6 SD). After birth, he developed tonic–clonic seizures and primary liver and kidney dysfunction. Newborn screening revealed hypothyroidism (TSH: 996.10 µU/ml; fT3: 0.13 ng/dl) and he received intravenous L-thyroxine. He required assisted ventilation. Furthermore, he showed generalized loss of subcutaneous fat with a striking progeroid appearance, hypertrichosis, arachnodactyly and adducted thumbs as well as facial dysmorphisms including low-set and large appearing ears, micro- and retrognathia, long, smooth philtrum, and wrinkled skin. He had congenital intestinal malrotation and needed surgery due to meconium ileus. After surgery, he developed renal failure and intracranial hemorrhage and died at the age of 12 days. Family history revealed two healthy older sisters, II.2 and II.3, and a brother who died few hours after birth (II.1). In addition, the mother had one early miscarriage (II.4).

### Identification of a homozygous *YRDC* mutation

Initial genetic testing for progeroid syndromes using a custom-designed multigene panel including 87 known disease-associated genes was unremarkable. Therefore, trio whole-exome sequencing (WES) was performed on DNA extracted from blood of the patient II.5 and his unaffected parents, I.1 and I.2, using the Agilent SureSelectXT Human All Exon V7 enrichment kit on an Illumina HiSeq4000 sequencer. We analyzed and filtered the trio WES data using the exome and genome analysis pipeline “Varbank 2.0” (https://varbank.ccg.uni-koeln.de/varbank2) of the Cologne Center for Genomics (CCG, University of Cologne, Germany), and we obtained a mean coverage of 60–65 and 96.1–96.3% of target were covered more than 10×. Trio-based WES is an effective diagnostic tool for identification of causative variants in individuals with developmental disorders (Palomares-Bralo et al. 2017). de novo variants are known to be one of the most common genetic causes of developmental disorders (Acuna-Hidalgo et al. 2016). Both parents of the index patient II.5 were unaffected, so our analysis included different modes of inheritance. We identified only a single heterozygous de novo variant, which was excluded as causative variant based on population frequency and lacking evidence for functional impairment on the affected protein (Supplementary Table S1). Based on the family history and the reported parental consanguinity, we next focused on homozygous variants consistent with a recessive transmission. We identified only one single homozygous variant not present in any current database of human genetic variations including the gnomAD (https://gnomad.broadinstitute.org) database (last access date 12/01/2021, (Karczewski et al. 2019)) and predicted to have a severe impact on protein structure. All de novo and rare biallelic or hemizygous variants are listed in the supplement (Supplemental Table S1). The homozygous missense variant, c.662T > C, was located in exon 4 of the *YRDC* gene (Fig. 1D) and leads to the substitution of an evolutionary highly conserved isoleucine at the amino acid position 221 by threonine (p.Ile221Thr). The variant was predicted as disease-causing by MutationTaster (http://www.mutationtaster.org), damaging by SIFT (https://sift.bii.a-star.edu.sg), probably damaging by PolyPhen‐2 (http://genetics.bwh.harvard.edu/pph2), and has a CADD (https://cadd.gs.washington.edu) score of 27.7, indicating deleteriousness of this variant. The mutation was embedded in the largest autosomal homozygous region (> 5 Mb) determined from the WES data set and was found in heterozygous state in both parents. Sanger sequencing was used to validate the WES data and to prove that the healthy siblings, II.2 and II.3, do not carry the variant in the homozygous state. DNA from the deceased brother was not available for genetic testing (Fig. 1B).

### Effects of the *YRDC* mutation on protein stability, localization and tRNA modification

To examine the cellular consequences of the p.Ile221Thr mutation on protein stability and expression levels, we compared the YRDC protein levels in primary dermal fibroblasts from patient II.5 and fibroblasts from a matched healthy control. Western blot analysis showed comparable expression levels of YRDC in both control and patient fibroblasts (Fig. 1E). Next, we analyzed the subcellular localization of wild type (WT) and mutant (MUT) YRDC by immunostaining. YRDC showed a widely diffused intracellular localization in both, WT and MUT cells (Fig. 1F). Taken together, these data suggest that the missense variant p.Ile221Thr in YRDC does not affect its stability or subcellular localization in patient fibroblasts.

YRDC has been described to be essential for the synthesis of t^6^A at position A37 of several tRNAs. To assess the effect of the mutation on the level of t^6^A in tRNAs, total RNA was extracted from patient’s skin fibroblasts and subjected to LC–MS/MS analysis. As expected, the global t^6^A level of the patient showed a significant decrease of 16.3% (Fig. 1G) compared to WT samples. This shows that the homozygous c.662T > C, p.Ile221Thr mutation in *YRDC* affects the t^6^A biosynthesis providing additional functional evidence for the causative nature of this homozygous missense mutation.

### Q-FISH-based evaluation of telomere length

**Fig. 2:**
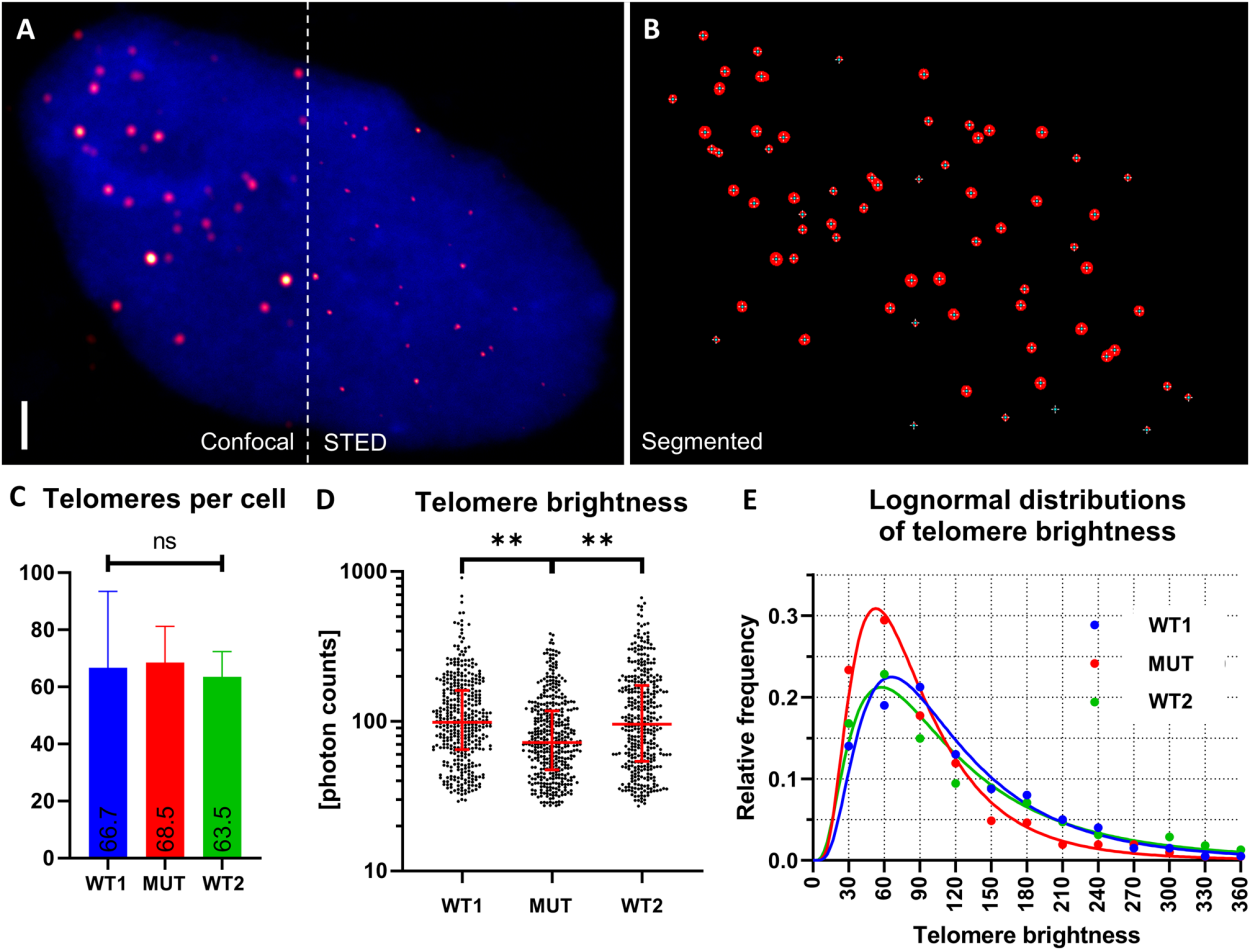
Q-FISH analysis of telomeres in patient fibroblasts and two controls. **A** STED imaging of a TelC-Star-635P FISH-staining (Red-hot LUT) with DAPI (blue, confocal) counterstain. *Z*-projection of STED imaging confirms that telomeres do not cluster and are distant enough to be resolved by confocal z-stacks. Scale bar = 2 µm. **B** Thresholder confocal image showing detected telomeres with marked center coordinates. Even partially overlapping spots could be resolved across a wide range of signal amplitudes. **C** Detection efficiency for telomeres is similar in all experimental groups despite telomere shortening, as shown by similar numbers of detected telomeres per cell after detection and fit process (*n* = 66.7 ± 26.8, 68.5 ± 12.7, 63.5 ± 8.9, mean ± std). **D** Telomere brightness is significantly reduced in patient fibroblasts (MUT). Each scatter includes the data of 6 nuclei. The nuclei of each respective sample had a mean (± std) telomere brightness of 126.3 ± 13.2 (WT1), 93.3 ± 14.4 (MUT), and 132.9 ± 22.0 (WT2) photon counts. Red lines indicate median and IQR. Stars indicate significance (*p* < 0.01) by nested one-way ANOVA of log-transformed data, sub grouped by nucleus. **E** Dark telomeres appear more frequently in MUT fibroblasts with altered genetic background. Telomere brightness data follows a lognormal probability function

It is well established that telomere shortening and aging-related processes are strongly associated with each other. However, the detailed mechanisms of, e.g., accelerated telomere shortening in monogenic progeria phenotypes are largely unknown. Telomere brightness by FISH staining has been validated as a solid measure of telomere lengths (Ourliac-Garnier and Londoño-Vallejo 2017) with a linear brightness-to-telomere relationship (Martens et al. 1998; Poon et al. 1999). We, therefore, established and performed a novel and highly sensitive in situ Q-FISH analysis in patient fibroblasts (MUT) relying on a 2-step procedure. First superresolution STimulated Emission Depletion (STED) microscopy was applied to in situ FISH samples (Fig. 2A) and used to confirm sufficient spatial separation of in situ FISH-labeled telomeres by at least 200 nm. As a second step, confocal imaging produced z-stacks of whole nuclei, and consequently individual telomeres were identified and analyzed automatically with image processing algorithms (Fig. 2B). Importantly, as a prerequisite for the comparative analysis, we have confirmed equal likelihoods of telomere detection in all samples regardless of average telomere brightness (Fig. 2C). Our analysis of telomere brightness revealed a significant decrease (*p* < 0.01) in telomere brightness in MUT as compared to an age-matched control (WT1) and a healthy adult (WT2) (Fig. 2D) with both matching numbers of passages. This corresponds to on average approximately 26% telomere shortening of chromosomes in patient cells, with shortening being seemingly proportional to regular individual telomere lengths. Notably, we observed a significant increase in the occurrence of short (detected as dark) telomeres (Fig. 2E). To further analyze whether this telomere shortening is associated with increased cellular senescence, we measured the senescence-associated beta-galactosidase activity in patient and control fibroblasts. We observed an increased number of beta-galactosidase-positive patient cells corresponding to a higher proportion of senescent cells (23.7% in patient cells compared to 14.5% in control fibroblasts), which in both cases could be elevated by approximately 15% by treatment with H_2_O_2_ (39.6% in patient cells compared to 28.7% in control fibroblasts) (Supplementary Figure S2).

### Specific gene expression signatures based on single-cell RNA sequencing (scRNA-seq)

**Fig. 3:**
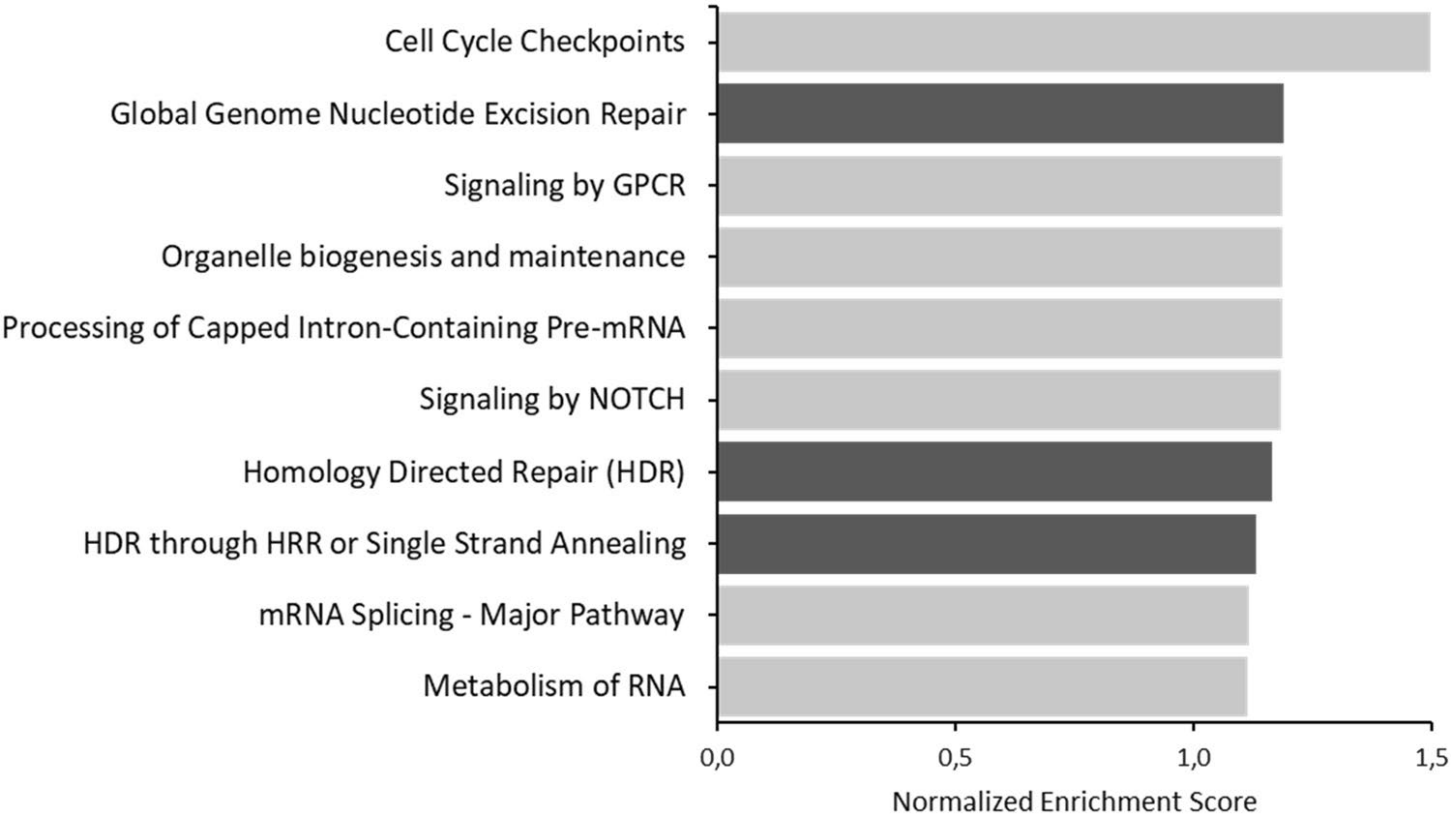
Whole transcriptome analysis based on single-cell RNA sequencing reveals different gene expression in YRDC-mutant fibroblasts. Gene Ontology (GO) and Reactome enrichment analysis showing the top 10 enriched pathways in patient fibroblasts compared to two sex- and age-matched controls. Categories related to DNA repair are highlighted in dark grey

ScRNA-seq can be a powerful tool for analyzing changes in gene expression at the cellular level and elucidating novel biological mechanisms. To explore the effect of the homozygous p.Ile221Thr mutation in YRDC on the transcriptome, we performed single-cell RNA sequencing on patient fibroblasts and two sex- and age-matched controls. We used Gene Set Enrichment Analysis (GSEA) to determine whether particular subsets of genes were differentially expressed. Of specific interest was the finding that among the top 10 enriched pathways in YRDC mutant cells we found “Global Genome Nucleotide Excision Repair”, “Homology Directed Repair” and “HDR through Homologous Recombination (HRR) or Single Strand Annealing (SSA)” (Fig. 3) to be altered. Even though the normalized enrichment scores were low, the prevalence of enriched pathways linked to DNA repair mechanisms suggests a possible interaction between YRDC function and maintenance of genomic stability. Genome instability is a cellular hallmark observed in cells of patients with different forms of premature aging syndromes, such as Cockayne syndrome, Ataxia–telangiectasia and Bloom syndrome (Wu and Hickson 2003; Trapp et al. 2007; Shiloh and Lederman 2017). Interestingly, YRDC has very recently been identified in CRISPR/Cas9 screens as important for DNA damage response in human cells (Olivieri et al. 2020).

### Altered DNA damage response (DDR) and DNA double-strand break (DSB) repair

**Fig. 4:**
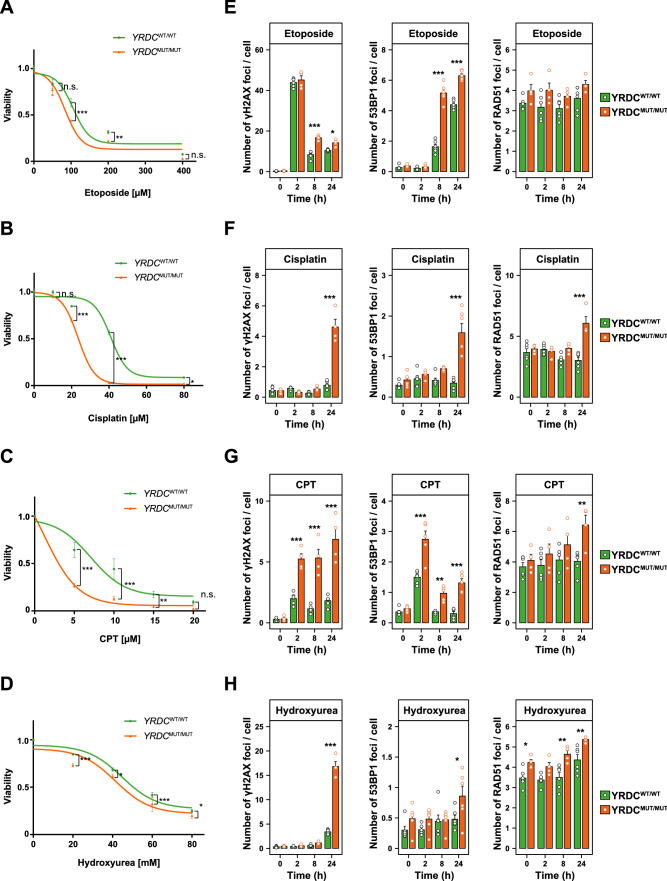
Homozygous c.662T > C mutation in *YRDC* confers cellular sensitivity to genotoxic agents and a DSB repair defect. **A–D** Cells of the indicated genotypes were treated with etoposide (**A**), cisplatin (**B**), CPT (**C**) or HU (**D**) for 96 h and viability was assessed by CellTiterGlo (CTG) assays. Error bars represent SD of the mean of three technical replicates with > 2000 cells/well. *p* values were calculated using *t* test with Welch’s correction not assuming equal variance. *p < 0.05, **p < 0.01, ***p < 0.001. **E–H** Cells of the indicated genotype were treated for 2 h with etoposide (150 µM) (**E**), cisplatin (20 µM) (**F**), CPT (2 µM) (**G**) or with HU (20 mM) (**H)** and stained for γH2AX (left panel), 53BP1 (middle panel) and RAD51 (right panel). Total number of γH2AX, 53BP1 and RAD51 foci per cell were quantified. Dots indicate means of separate replicates; *n* = 3 wells; > 2000 cells/well; experiments were independently performed three times; two-way ANOVA with Bonferroni’s test. *p < 0.05, **p < 0.01, ***p < 0.001

To gain further insight into whether different pathways involved in DDR and DNA repair are affected by the identified mutation in *YRDC*, we next examined the response of patient’s dermal fibroblasts to different genotoxic agents. MUT and WT fibroblasts were treated with etoposide, cisplatin, camptothecin (CPT) or hydroxyurea (HU) and cell viability was determined for different timepoints after treatment (Fig. 4A–D). Interestingly, MUT fibroblasts showed an increased susceptibility to all employed genotoxic agents compared to WT controls, with the strongest effect observed after treatment with cisplatin or CPT (Fig. 4B, C), possibly suggesting impaired DNA DSB repair in MUT cells (Dasari and Tchounwou 2014).

To further characterize involved DNA repair pathways in YRDC mutant cells, we employed automated immunofluorescence microscopy to quantify nuclear γH2AX foci, as a surrogate for unrepaired DNA lesions, as well as 53BP1 and RAD51 foci, which are markers for non-homologous end joining (NHEJ) and homologous recombination (HRR) pathways of DSB repair, respectively (Jachimowicz et al. 2019). Without treatment, we did not detect any significant differences in the numbers of γH2AX, 53BP1, and RAD51 foci in MUT compared to WT cells. In response to etoposide, we observed a significantly delayed clearance of γH2AX foci in MUT compared to WT cells (Fig. 4E, left panel). In contrast to the etoposide response, MUT cells treated with cisplatin, CPT or HU showed a continuous increase in γH2AX foci throughout the 24-h observation period compared to WT cells (Fig. 4F–H, left panel). Furthermore, MUT cells generally displayed higher numbers of 53BP1 foci following treatment with all four genotoxic agents compared to WT cells (Fig. 4E–H, middle panel). This difference was especially pronounced 24 h after treatment coinciding with high levels of γH2AX foci in MUT cells. With the exception of etoposide-treated cells, MUT cells also displayed increased numbers of RAD51 foci following DNA damage induction (Fig. 4E–H, right panel). Corresponding immunofluorescence microscopy images are included in the supplement (Supplemental Figures S3–S5). Taken together, these results combined with the ScRNA-seq data may suggest that the homozygous p.Ile221Thr mutation in YRDC confers hypersensitivity to DNA-damaging agents and leads to a global DNA DSB repair defect, potentially affecting both HRR and NHEJ.

## Discussion

Here, we present a patient with a severe developmental disorder with progeroid features caused by a novel homozygous missense mutation in *YRDC*. Our functional studies indicate that mutant YRDC results in defective tRNA modification, telomere shortening and increased genomic instability, thereby, giving first insights into the molecular pathogenesis of this novel accelerated aging phenotype.

YRDC is a highly conserved protein interacting with the well-described KEOPS complex (Lin et al. 2018). Mutations in genes encoding subunits of the KEOPS complex cause Galloway–Mowat syndrome (GAMOS, OMIM#251300), a rare autosomal recessive condition characterized by the association of early onset nephrotic syndrome and microcephaly with central nervous system anomalies (Braun et al. 2017). Although patients with mutations in KEOPS share some phenotypic features with the individual reported in our study, such as, e.g., renal abnormalities or seizures, progeroid features as presented by our patient were not observed in GAMOS patients with mutations in KEOPS complex components. A recently published study reported a family with an affected child carrying compound heterozygous *YRDC* mutations (missense mutation (c.251C > T, p.Ala84Val) and a 4-base pair deletion leading to a frameshift (c.721_724del, p.Val241Ilefs*72)) and a further family with two affected children carrying a homozygous in-frame deletion of leucine 265 (c.794_796del, p.Leu265del) (Arrondel et al. 2019). Similar to our patient, all these individuals died early (oldest age of death 15 months) and they shared some additional specific clinical features, such as congenital hypothyroidism, renal failure, and facial dysmorphism. Like our patient, the two affected siblings reported by Arrondel et al*.* showed arachnodactyly and primary microcephaly, while these features were not present in the single patient of the cited study. Arrondel et al*.* (2019) suggested that mutations in *YRDC* cause an “extremely severe form of GAMOS”. The main clinical features are summarized in Table 1. Considering the phenotypic overlap of all four patients, we postulate that patients with homozygous or compound heterozygous *YRDC* mutations should be viewed as a distinct entity based on the specific phenotypic features of congenital hypothyroidism, progeroid appearance and premature death.

**Table 1:** Summary of the clinical findings in our patient and the affected individuals with biallelic variants in *YRDC* reported by Arrondel et al. (2019)

	Reported here	Arrondel et al. (2019)		
Family	A	B	C	
ID	Patient 1	Patient 2	Patient 3	Patient 4
Variant	c.662T > C; (p.Ile221Thr)	c.251C > T/c.721_724del; (p.Ala84Val)/(p.Val241Ilefs*72)	c.794_796del; (p.Leu265del)	c.794_796del; (p.Leu265del)
Zygosity	Homozygous	Compound heterozygous	Homozygous	Homozygous
Exon	4	1/4	4	4
Gender	Male	Female	Male	Female
Ancestry	Northern Iraq	European	European	
Consanguinity	Distantly	No	Yes	
Clinical manifestations				
Birth	Born at 37 + 0 weeks	ND	ND	ND
Birth length	46 cm (− 1.8 SD)	ND	ND	ND
Birth weight	1740 g (− 3.1 SD)	ND	ND	ND
Head circumference at birth	29 cm (− 3.6 SD)	ND	ND	ND
Seizures	+	−	−	−
Kidney dysfunction	+	+	+	+
Liver dysfunction	+	−	−	−
Hypothyroidism	+	+	+	+
Facial dysmorphisms	Progeroid appearance, low-set and large appearing ears, micro- and retrognathia, long, smooth philtrum, and wrinkled skin	+	+	+
Brain anomaly	Primary microcephaly	Secondary microcephaly	Primary microcephaly	Primary microcephaly
Skeletal	Arachnodactyly, adducted thumbs	−	Arachnodactyly	Arachnodactyly
Intestinal malrotation	+	−	−	−
Age at death	12 days	15 months	1.5 months	3 months

+ Present, − Absent, *ND* Not documented, *SD* Standard deviation

Our analysis of the functional consequences of the identified p.Ile221Thr mutation in YRDC indicated that mutant YRDC might result in decreased threonylcarbamoylation of the N6 nitrogen of the adenosine at the tRNA position 37 (t^6^A). Previous studies have shown that YRDC acts in this process together with the KEOPS complex, and mutations in KEOPS proteins as well as in YRDC have been linked to decreased t^6^A levels (El Yacoubi et al. 2009; Srinivasan et al. 2011; Perrochia et al. 2013; Lin et al. 2018). The reduction of t^6^A modifications that we observed in patient-derived fibroblasts strongly supports causality of the identified missense variant p.Ile221Thr in YRDC. Still, based on the different phenotypic presentation of GAMOS patients with mutations in KEOPS proteins and patients with YRDC mutations, it is likely that YRDC is involved in additional cellular processes which are not depending on the KEOPS complex.

As we observed progeroid features in our patient, we focused on the putative relevance of YRDC in aging processes, reflected by its role in telomere maintenance and DDR. Arrondel et al*.* (2019) reported that the telomere length in their patients with YRDC mutations was not affected. Their results were based on a telomere restriction fragment assay and traditional Southern blot analysis, which revealed a higher telomere length in affected children compared to their parents. A solid method to measure the telomere length is Q-FISH analysis (Martens et al. 1998; Poon et al. 1999; Ourliac-Garnier and Londoño-Vallejo 2017). Here, we performed a novel and highly sensitive Q-FISH analysis to investigate the impact of mutant YRDC on telomere maintenance. Performing three-dimensional (3D) confocal imaging with 200 nm axial step size followed by 3D image analysis we were able to circumvent most of the inherent disadvantages of conventional Q-FISH methods, such as, e.g., insufficient separation of adjacent and superimposed telomeres and frequent out-of-focus detections (Poon et al. 1999). We were able to detect and quantify most individual telomeres in the appropriate respective imaging planes and thus obtained a remarkable cellular telomere coverage of over 70%. This confirms the strength of our 3D imaging and analysis approach, resulting in more reliable and accurate readout of individual telomere brightness. Our analysis revealed a significant decrease in telomere brightness in patient’s fibroblasts, indicating significant telomere shortening in the patient cells. This is the first time that microscopy-based Q-FISH analysis of individual telomeres was used to determine telomere shortening in a human progeroid phenotype. Importantly, our data confirms that telomeres were detected at same efficiencies in all groups regardless of the average telomere brightness, confirming the robustness of this approach to identify telomeres individually in three dimensions. In contrast to the study by Arrondel et al*.,* we compared telomere length to an age-matched control instead of parental control samples, which might explain the differences in the age-associated outcome of the analyses.

However, the exact mechanistic link between YRDC and telomere length remains to be elucidated in comprehensive future studies and rescue experiments are required to confirm our findings. Some previous studies have speculated that the influence of YRDC/Sua5 and the KEOPS complex on telomere length might be attributed to the lack of t^6^A modification (Daugeron et al. 2011; El Yacoubi et al. 2011; Perrochia et al. 2013; Huang et al. 2019). In contrast, Downey et al*.* (2006) hypothesized that the KEOPS complex promotes both telomere uncapping and telomere elongation. Meng et al*.* (2009, 2010) showed that SUA5 also positively regulates telomere maintenance. Liu et al*.* (2018) supported with their results a model in which Sua5 and the KEOPS complex are essential for telomere homeostasis independent of its t6A biosynthesis activity and suggested that KEOPS regulates telomere length by promoting G-overhang generation. Although its precise action is still elusive, our data provides additional support for the assumption that YRDC is linked to molecular mechanisms underlying accelerated aging processes.

In addition, we analyzed the senescence-associated β-galactosidase activity and observed that patient-derived fibroblasts show an increased number of β-galactosidase-positive cells corresponding to a higher proportion of senescent cells. Cellular senescence is defined as a stable state of cell cycle arrest (Rhinn et al. 2019). Besides the well-established role of accumulating senescent cells in aging and age-associated conditions, senescence has been linked to proper embryonic development (Gal et al. 2021), and positive senescence-associated β-galactosidase staining can be observed at multiple locations in whole-mount mice embryos (Muñoz-Espín et al. 2013). Recent studies confirmed that cellular senescence contributes to normal tissue patterning and organ development during embryogenesis and later in life is essential for tissue repair and regeneration (Gal et al. 2021; Di Micco et al. 2021; Muñoz-Espín und Serrano 2014). Accumulation of senescent cells might directly impair tissue proliferation (Rhinn et al. 2019) and thereby contribute to the severe developmental anomalies observed in our patient.

Furthermore, ScRNA-seq revealed an upregulation of DDR pathways possibly indicating genomic instability in YRDC mutant fibroblasts. Keeping in mind that enrichment scores were low, these results need to be interpreted carefully. For validation, we performed additional functional assays using patient fibroblasts. This further analysis showed a generally increased susceptibility to DNA-damaging agents and a global DNA DSB repair defect, affecting both HRR and NHEJ. Impairment of DDR mechanisms is commonly observed in patients with different progeroid syndromes including, e.g., Hutchinson–Gilford progeria syndrome, Werner syndrome, or Cockayne syndrome. Interestingly, Braun et al*.* (2017) observed increased activation of DDR pathways resulting in reduced cellular viability after knockdown of KEOPS complex subunits, indicating that YRDC might contribute to DDR in interaction with the KEOPS complex. Our results are consistent with the very recently published CRISPR/Cas9 screen, in which Olivieri et al. (2020) showed as well that YRDC might be an important factor for DNA damage response and thereby promoting DNA integrity. Further investigations are required to verify this hypothesis.

In summary, our data provides evidence that biallelic mutations in *YRDC* result in a severe developmental disorder with progeroid features and might be linked to reduced levels of t6A modifications as well as telomere shortening and alteration of DNA damage response.

## Methods

### Whole-exome sequencing

Whole-exome sequencing on DNA of the patient II.5 and both parents was performed using the Agilent SureSelectXT Human All Exon V7 enrichment kit on an Illumina HiSeq 4000 sequencer. The “Varbank 2” pipeline of the Cologne Center for Genomics (CCG) was used to analyze the exome data using the following filter criteria: coverage of > 6 reads, quality score of > 10, allele frequency ≥ 25%, and a minor allele frequency (MAF) < 0.1% in the gnomAD (https://gnomad.broadinstitute.org) database. The following databases were used to obtain gene information: National Center for Biotechnology Information (NCBI; https://www.ncbi.nlm.nih.gov), Ensembl Genome Server (http://www.ensembl.org), UCSC Genome Bioinformatics (http://genome-euro.ucsc.edu) and Genome Aggregation Database (gnomAD; http://gnomad.broadinstitute.org). The variant identified by whole-exom sequencing was amplified from DNA of the index patient and PCR products were sequenced by BigDye Terminator method on an ABI 3500XL sequencer (Life Technologies, Germany). The identified mutation was re-sequenced in an independent experiment and tested for co-segregation within the family.

### Cell lines and cell cultures

Primary dermal fibroblasts established from patient II.5 and respective controls were cultured in Dulbecco’s modified Eagle medium (DMEM, Gibco) supplemented with 10% fetal calf serum (FCS, Gibco), and antibiotics. All functional assays presented in the manuscript were performed using cell lines that were matched for passage number and, where possible, age as well as sex.

### Protein isolation and analysis

Primary dermal fibroblasts were solubilized using ice-cold RIPA buffer (10 mM Tris, pH: 8.0; 150 mM NaCl; 1 mM EDTA; 1% NP-40; protease inhibitors P 2714 [Sigma-Aldrich, USA]). The total protein concentration of extracts was determined using the BCA Protein Assay Kit (Thermo Fisher Scientific, USA). 15–25 µg of total cell lysates were separated by Mini-PROTEAN^®^ TGX™ Precast Gels (Bio-Rad, Germany) and blotted onto nitrocellulose membranes (Bio-Rad, Germany). Protein detection was performed using an antibody to YRDC (Santa Cruz Biotechnology Inc., USA). Secondary antibodies conjugated to peroxidase (Santa Cruz Biotechnology Inc., USA) were used and blots were developed using an enhanced chemiluminescence system, Clarity Max Western (Bio-Rad, Germany), followed by detection using the ChemiDoc™ Touch Imaging System (Bio-Rad, Germany).

### Immunofluorescence stainings and fluorescence measurements

For immunofluorescence detection of YRDC, primary dermal fibroblasts were grown on coverslips, washed with PBS, fixed with 4% paraformaldehyde for 15 min and permeabilized with 1% Triton X-100 for 10 min. Upon blocking for 30 min with 3% BSA in PBS, slides were incubated with YRDC antibodies (1:500, Santa Cruz Biotechnology Inc) overnight. Slides were washed with PBS and incubated with Alexa Fluor® 488 conjugated goat–anti-rabbit antibodies (Thermo Fisher Scientific) for 1 h. Subsequently, slides were washed with PBS, mounted in Vectashield with DAPI (H 1200; Vector Laboratories) and viewed with an Olympus FLUOVIEW FV1000 confocal laser scanning microscope.

For automated immunofluorescence microscopy, human dermal fibroblasts were seeded onto 96-well plates. For RAD51 stainings, cells were additionally pre-extracted in sucrose buffer (25 mM HEPES pH 7.5, 50 mM NaCl, 1 mM EDTA, 3 mM MgCl_2_,300 mM sucrose, 0.5% Triton-X) for 2 min on ice. Cells were fixed for 10 min in room temperature with 4% paraformaldehyde, followed by three washes with ice-cold 1 × PBS. Subsequently, cells were blocked for 60 min at room temperature in PBS containing 5% normal goat serum (NGS), 2% bovine serum albumin and 0.01% Triton X-100. Incubation with the primary antibody was performed over night at 4 °C. Incubation with the secondary antibody was performed for 1 h at room temperature. Analysis was performed as described below in “High-content screening microscopy.”

### Quantification of t^6^A modification

500 ng of total RNA per sample was digested to nucleosides using 0.6 U nuclease P1 from *P. citrinum* (Sigma-Aldrich), 0.2 U snake venom phosphodiesterase from *C. adamanteus* (Worthington), 2 U FastAP (Thermo Scientific), 10 U benzonase (Sigma-Aldrich), 200 ng Pentostatin (Sigma-Aldrich) and 500 ng Tetrahydrouridine (Merck-Millipore) in 25 mM ammonium acetate (pH 7.5; Sigma-Aldrich) over night at 37 °C. The nucleosides were then spiked with internal standard (^13^C stable isotope-labeled nucleosides from *S. cerevisiae*) and subjected to analysis. 100 ng of digested RNA and 50 ng internal standard were analyzed via LC–MS [Agilent 1260 series and Agilent 6460 Triple Quadrupole mass spectrometer equipped with an electrospray ion source (ESI)]. The solvents consisted of 5 mM ammonium acetate buffer (pH 5.3; solvent A) and LC–MS grade acetonitrile (solvent B; Honeywell). The elution started with 100% solvent A with a flow rate of 0.35 ml/min, followed by a linear gradient to 8% solvent B at 10 min and 40% solvent B after 20 min. Initial conditions were regenerated with 100% solvent A for 10 min. The column used was a Synergi Fusion (4 µM particle size, 80 Å pore size, 250 × 2.0 mm; Phenomenex). The UV signal at 254 nm was recorded via a diode array detector (DAD) to monitor the main nucleosides. ESI parameters were as follows: gas temperature 350 °C, gas flow 8 l/min, nebulizer pressure 50 psi, sheath gas temperature 350 °C, sheath gas flow 12 l/min, capillary voltage 3000 V. The MS was operated in the positive ion mode using Agilent MassHunter software in the dynamic MRM (multiple reaction monitoring) mode. For relative quantification, an internal calibration was applied as described previously (Kellner et al. 2014).

### Single-cell RNA sequencing (scRNA-seq)

A full-length, single-cell RNA-seq approach was used with the ICELL8 Single-Cell System. Cell suspensions were fluorescently labeled with live/dead stain (DAPI and Texas Red) for 15 min prior to their dispensing into the 5184 microchip nanowells (ICELL8 System). After selection of wells containing single, live cells, cDNA was synthesized via oligo-dT priming in a one-step RT-PCR reaction. P5 indexing primers for subsequent library preparation were indexed into all wells, with each of the 72 rows on the ICELL8 chip receiving a different index, in addition to Terra polymerase and reaction buffer. Transposition enzyme and reaction buffer (Tn5 mixture) were dispensed to selected wells, and the transposition reaction was performed. P7 indexing primers were dispensed to wells, with each of the 72 columns on the chip receiving a different index. Final Illumina libraries were amplified and pooled as they are extracted from the chip. Pooled libraries were purified and size selected using Agencourt AMPure XP magnetic beads (Beckman Coulter) to obtain an average library size of 500 bp. A typical yield for a library comprised of ~ 1300 cells was ~ 15 nM. Libraries were sequenced on the HiSeq 4000 (Illumina) to obtain on average ~ 0.5 Mio reads per cell (SE; 50 bp).

### Data pre-processing and quality control

Raw sequencing files (bcl) were demultiplexed and fastq files were generated using Illumina bcl2fastq software (v2.20.0). For data pre-processing, the ICELL8 mappa analysis pipeline (version 0.9) was run. In brief, mappa_demuxer was used to allocate the reads to the cells based on the cell barcodes provided in the well-list file. Subsequently, the mappa_analyser was used as a wrapper function to perform read trimming with cutadapt (version 2.5), genome alignment to Homo sapiens genome GRCh38 using STAR (version 2.7.2b), read counting for exonic, genomic and mitochondrial regions using featureCounts (version 1.6.4) and summarization using the R package hanta (version 1.0.0). As part of the hanta package, the final matrix of reads counts for all genes in all cells underwent quality control (QC) filtering for low expressions using default parameters: (a) for cells, only those with at least 10,000 reads associated to at least 300 different genes were kept, and (b) for genes, only those containing at least 100 reads mapped to them from at least 3 different cells were kept. After applying these quality control filtering criteria, we analyzed 201 out of 256 single patient cells and 428 out of 614 single cells of the two sex- and age-matched controls. The de-regulated genes (abs(log2FolgChange) > 0.25) are listed in the supplement (Supplemental Table S2).

### Data analysis

For differential expression (DE) analysis between pairs of samples, the QC-filtered read count matrix was used as input to determine which genes were differentially expressed using Wilcoxon Rank Sum and Signed Rank Tests. For the individual DE results, a rank score was calculated for each gene using the formula rank = − log10(*p*-value) × log2FC, where *p* value is the raw *p* value and log2FC is the log2 fold-change. The ranks were used as input for an enrichment analysis using WebGestaltR (version 0.4.4) for all gene ontologies and Reactome pathways.

### Cell viability measurement

Cell lines were plated into 384-well plates at densities of 5000 cells/well. 24 h later, cells were treated with various doses of genotoxic chemicals for 96 h. After incubation, room-temperature CellTiter-Glo® Reagent (Promega, USA) was added 1:1 to each well and the plates were incubated at room temperature for 10 min. Luminescence was measured with the Tecan Infinite M1000 Pro (Tecan, Männedorf, CHE) and normalized against control cells treated with vehicle solution. Error bars represent SD of the mean of three independent experiments. *p* values were calculated using *t* test with Welch’s correction not assuming equal variance. *p < 0.05, **p < 0.01, ***p < 0.001.

### High-content screening microscopy

High-content screening (HCS) microscopy was performed on human dermal fibroblasts using a Thermo Fisher Cellomics ArrayScan XTI with LED light source. Images of 1104 × 1104 pixels were acquired with a 20 × objective (Zeiss) and analyzed using the Cellomics software package (Colocalization V.4 Bioapplication). Images were background corrected (3D surface fitting) and DAPI stained cell nuclei were identified according to the object selection parameters size: 100–1500 μm^2^, ratio of perimeter squared to 4π area: 1–2, length-to-width ratio: 1–5, average intensity: 400–4000, total intensity: 4 × 10^6^–2 × 10^7^. Foci of γH2AX, 53BP1 and RAD51 were quantified within the nuclear region at another excitation wavelength (485 ± 20 nm or 650 ± 20 nm). Object selection parameters for foci were size: 0.1–10 μm^2^, ratio of perimeter squared to 4π area: 1–5, length-to-width ratio: 1–5, average intensity: 500–10,000, total intensity: 300–2 × 10^6^. HCS microscopy results are means ± standard deviation; > 2000 cells/well; three technical replicates were measured per experiment; two-way ANOVA with Bonferroni’s test. *p < 0.05, **p < 0.01, ***p < 0.001.

### Q-FISH-based evaluation of telomere length from primary dermal fibroblasts

For preparation of cells for Q-FISH analysis, primary cell cultures of human dermal fibroblasts were maintained in 75TC flasks up to passage 5, then split and harvested 48 h later at approximately 75% confluency. Therefore, cells were trypsinized for 1–2 min, trypsin was neutralized by adding media, and cells were pelleted (5 min, 200 g). Supernatants were completely removed and 10 ml of pre-warmed (37 °C) hypotonic solution (0.0025 M Na citrate, 0.04 M KCl) was dropwise added to pellets while vortexing slowly. Cells were incubated for 15 min at 37 °C and centrifuged (5 min, 200 g, room temperature). Supernatants were partly removed leaving approximately 1 ml in the tubes.

For fixation, cell pellets slowly resuspended by vortexing. 10 ml of cold Carnoy fixative (75% methanol, 25% acetic acid) was added dropwise to the pellets while vortexing with increasing speed. Then cells were pelleted at (5 min, 200 g, room temperature) and supernatants removed leaving approximately 1 ml in the tubes. The fixation/centrifugation steps were repeated three times. Cells were resuspended in a small volume of fixative and cell suspension dropped onto clean superfrost slides at optimal ambient humidity. Slides were air-dried and spreading efficiencies were checked by light microscopy. Slides with cell spreads were dried overnight.

Pretreatment slides were rehydrated for 15 min in PBS, fixed in 4% formaldehyde in PBS for 4 min at 37 °C, washed twice in PBS for 5 min at 37 °C and air-dried. 100 μl of RNase A solution (100 µg/ml in 2 × SSC) per slide was added and slides were incubated for 1 h at 37 °C in a Thermobrite incubator (a programmable temperature controlled slide processing system for FISH procedures). Afterwards the slides were washed three times in 2 × SSC and once in distilled water. The slides were immersed in 0.005% Pepsin for 4 min at 37 °C for protein digestion, washed twice in PBS for 3 min at 37 °C and fixed in 4% formaldehyde in PBS for 4 min at 37 °C. The slides were washed twice in PBS for 5 min at 37 °C (× 2) and once in PBS for 5 min at room temperature. Afterwards the slides were sequentially dehydrated for 2 min in 70%, 85%, 100% ethanol, respectively, and air-dried.

### Denaturation and PNA hybridization

For denaturation and PNA hybridization, slides were placed in a pre‐heated Thermobrite at 80 °C for 5 min. 100 μl of pre-heated PNA probe (TelC Star635P, 200 nM, Eurogentec) in hybridization solution (Oxford Gene Technology) was distributed per slide, overlaid with a coverslip and denatured at 80 °C for 5 min in the dark. The slides were placed in a humidified hybridization chamber at 4 °C overnight for PNA probe hybridization (the coverslips were fixed with silicon glue).

Afterwards, slides were immersed in washing solution (2 × SSC, 1% Tween-20) at room temperature to remove the coverslip and silicon glue and washed twice in washing solution for 10 min at 55–60 °C, afterwards washed twice in 2 × SSC and once in distilled water. After drying on air in the dark a drop of mounting media (Prolong Gold Antifade Reagent with DAPI, Invitrogen) was put onto slide, the slide was carefully covered with a coverslip and incubated in the dark overnight.

### Q-FISH microscopy

FISH slides were imaged in confocal and STED mode using a custom-built setup based on an Olympus IX83 inverted microscope with an Abberior QUAD scanner. Slides were mounted on a 100 × 1.4 NA oil-immersion objective and intact nuclei were identified in the DAPI channel. DAPI and TelC-Star635P signals were excited at 405 nm and 633 nm excitation wavelengths and recorded with 422–467 nm and 650–720 nm emission filters, respectively. 3D-Confocal imaging was performed at 80 nm × 80 nm–200 nm pixel size (*x*,*y*,*z*) at 100 µs pixel dwell time. In addition, single frames of imaging planes including bright TelC-Star635P signal spots were acquired using STED mode to confirm spatial separation of telomeres in confocal imaging.

### Q-FISH image analysis

In ImageJ Fiji, Images were manually masked by the DAPI signal to exclude background signals outside nuclei. TelC-Star635P signals were smoothed by a Gaussian filter with *σ* = 80 nm and subjected to a 3D spot detection routine (Herbert et al. 2014) with a background threshold of 25 counts and a minimum spot size of 10 voxels. The retrieved center of mass coordinates for each telomere spot were used to center two orthogonal lateral fits of the signal by a Gaussian function. The better-fit result was used to retrieve telomere brightness from its amplitude, when at least one fit was sufficient (*r*^2^ > 0.8). No further image or data filtering was applied.

## Supplementary Information

Below is the link to the electronic supplementary material.

Supplementary file1 (DOCX 311 KB)

Supplementary file2 (DOCX 400 KB)

Supplementary file3 (AI 6764 KB)

Supplementary file4 (AI 4859 KB)

Supplementary file5 (AI 9836 KB)

Supplementary file6 (XLSX 11 KB)

Supplementary file7 (XLSX 201 KB)

## Data Availability

The data that support the findings of this study are available on request from the corresponding author. The data are not publicly available due to privacy or ethical restrictions.
