# Supplementary material for: Biallelic variants in YRDC cause a developmental disorder with progeroid features
Source: Hum Genet. 2021 Sep 20;140(12):1679–93. doi: 10.1007/s00439-021-02347-3 (PMC8553732; doi:10.1007/s00439-021-02347-3)
Supplement: Supplementary file 1 — Supplementary file1 (DOCX 311 KB) [file 439_2021_2347_MOESM1_ESM.docx]

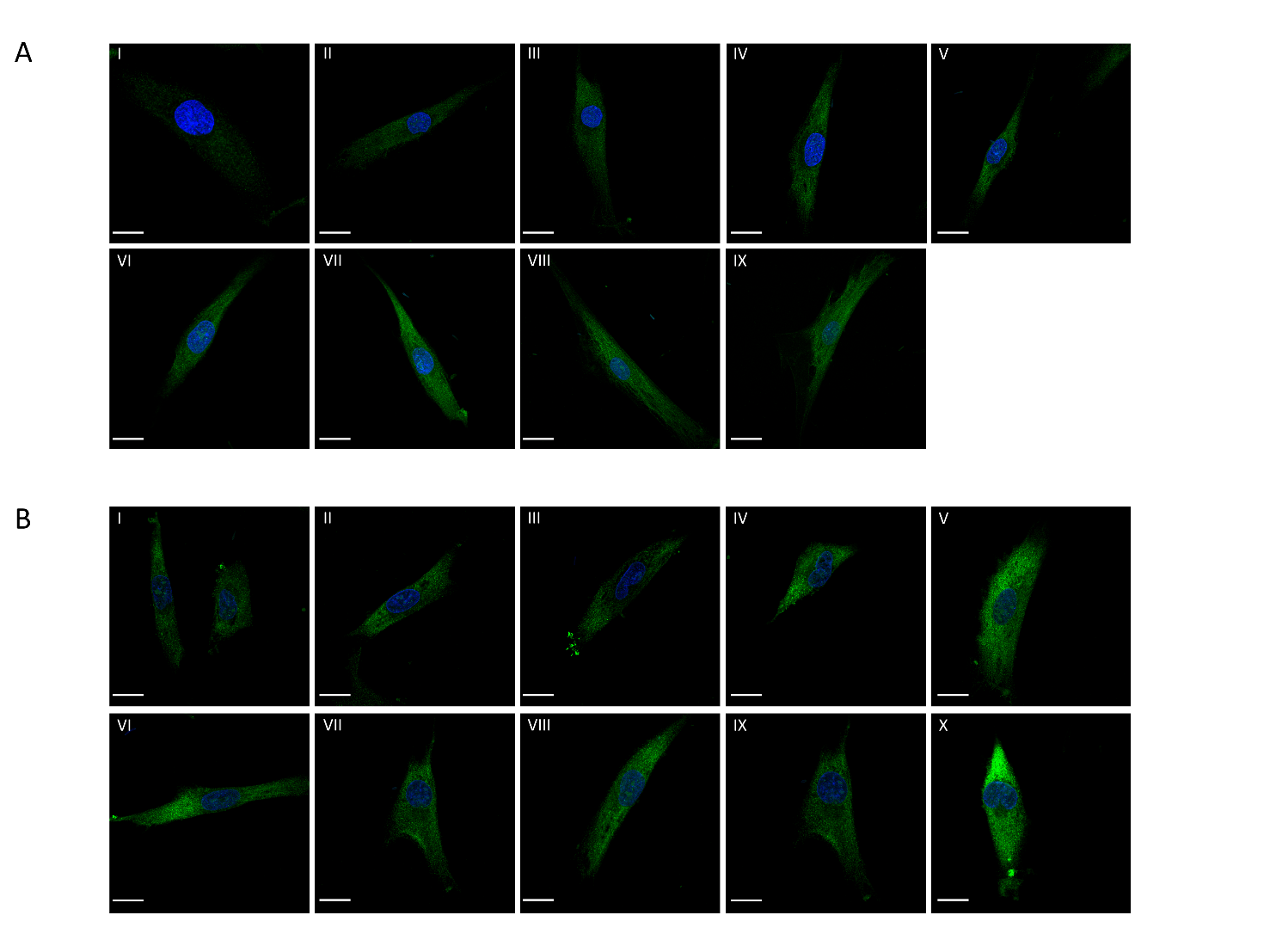


**Supplementary Figure S1:** Analysis of subcellular localization of WT (A) and mutant (B) YRDC by immunofluorescence staining in control (A) and patient-derived (B) fibroblasts using anti-YRDC-antibodies (green). Nuclei were counterstained with DAPI (blue). Scale bar, 20 µm.
