# Supplementary material for: Biallelic variants in YRDC cause a developmental disorder with progeroid features
Source: Hum Genet. 2021 Sep 20;140(12):1679–93. doi: 10.1007/s00439-021-02347-3 (PMC8553732; doi:10.1007/s00439-021-02347-3)
Supplement: Supplementary file 2 — Supplementary file2 (DOCX 400 KB) [file 439_2021_2347_MOESM2_ESM.docx]

**
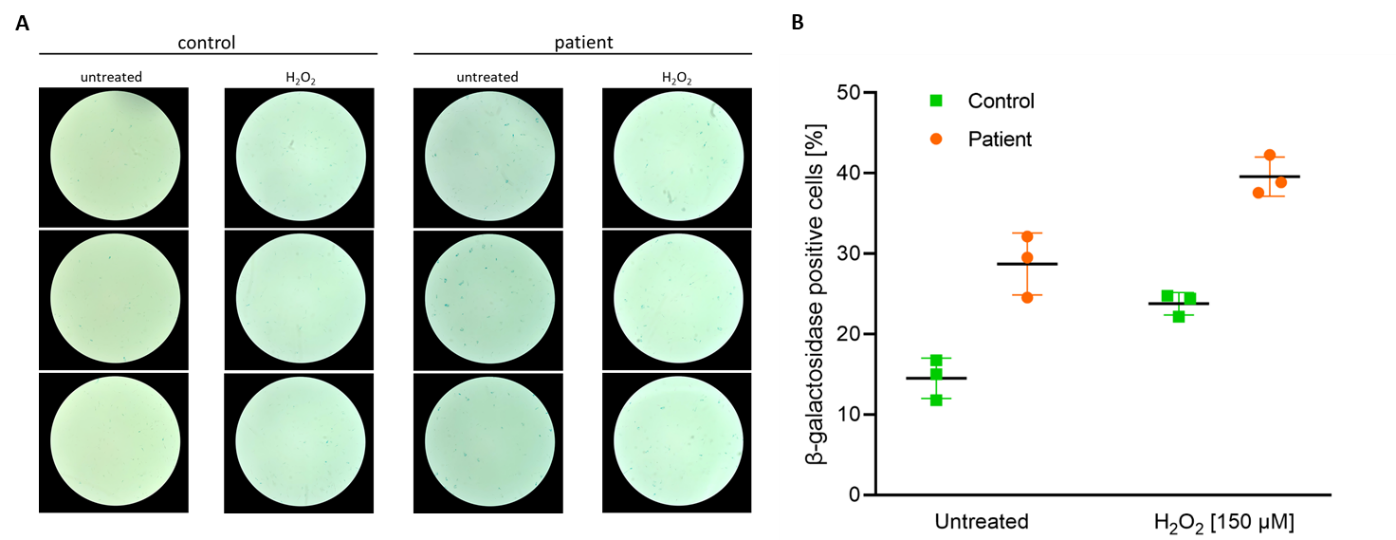
**

**Supplementary Figure S2:** Senescence associated β-galactosidase staining of control and patient fibroblast. **A** Cells were treated with 150 µM H2O2, and β-galactosidase positive cells shown by blue granules within the cytoplasm were counted using a bright-field microscope. Technical triplicates have been performed on one biological sample each, and 500 to 800 cells were counted for each sample and triplicate. Three representative pictures for each sample and condition are shown. **B** Analysis of senescence-associated β-galactosidase activity in patient and control fibroblasts, control: green squares, patient: orange dots.

**Supplementary Methods**

**β-galactosidase assay**

Cell lines were seeded in 6-well plates at densities of 10000 cells/well. 24 h later, cells were either untreated or treated for 1 h with 150 μM H2O2. After incubation, cells were washed with PBS and cultured 23 h in proliferation medium. β-galactosidase activity was measured using the senescence β-Galactosidase staining kit (Cell Signaling Technology, USA) according to manufacturer’s protocol, and senescence-associated β-galactosidase activity was determined by counting cells in randomly chosen parts of the wells under a bright-field microscope.
